# Proteomic analysis of tylosin-resistant *Mycoplasma gallisepticum* reveals enzymatic activities associated with resistance

**DOI:** 10.1038/srep17077

**Published:** 2015-11-20

**Authors:** Xi Xia, Congming Wu, Yaowen Cui, Mengjiao Kang, Xiaowei Li, Shuangyang Ding, Jianzhong Shen

**Affiliations:** 1 Beijing Key Laboratory of Detection Technology for Animal-Derived Food Safety, China Agricultural University, Beijing 100193, People’s Republic of China; 2 Key Laboratory of Detection for Veterinary Drug Residue and Illegal Additive, Ministry of Agriculture, Beijing 100193, People’s Republic of China; 3 College of Veterinary Medicine, China Agricultural University, Beijing 100193, People’s Republic of China; 4 China Institute of Veterinary Drugs Control, Beijing 100081, People’s Republic of China

## Abstract

*Mycoplasma gallisepticum* is a significant pathogenic bacterium that infects poultry, causing chronic respiratory disease and sinusitis in chickens and turkeys, respectively. *M. gallisepticum* infection poses a substantial economic threat to the poultry industry, and this threat is made worse by the emergence of antibiotic-resistant strains. The mechanisms of resistance are often difficult to determine; for example, little is known about antibiotic resistance of *M. gallisepticum* at the proteome level. In this study, we performed comparative proteomic analyses of an antibiotic (tylosin)-resistant *M. gallisepticum* mutant and a susceptible parent strain using a combination of two-dimensional differential gel electrophoresis and nano-liquid chromatography-quadrupole-time of flight mass spectrometry. Thirteen proteins were identified as differentially expressed in the resistant strain compared to the susceptible strain. Most of these proteins were related to catalytic activity, including catalysis that promotes the formylation of initiator tRNA and energy production. Elongation factors Tu and G were over-expressed in the resistant strains, and this could promote the binding of tRNA to ribosomes and catalyze ribosomal translocation, the coordinated movement of tRNA, and conformational changes in the ribosome. Taken together, our results indicate that *M. gallisepticum* develops resistance to tylosin by regulating associated enzymatic activities.

*Mycoplasma gallisepticum* is a bacterium that causes chronic respiratory disease in chickens and sinusitis in turkeys. *M. gallisepticum* infection can lead to considerable economic losses in the poultry industry because it results in reduced weight gain and egg production, as well as increased embryo mortality, in commercial birds^1^. Macrolides, such as tylosin, are a class of antibiotics that are important for the clinical treatment of *M. gallisepticum* infection. However, long-term and often incorrect use of macrolides has resulted in increased *M. gallisepticum* resistance, which has reduced the clinical efficacy of the drugs^2^,^3^,^4^,^5^.

Macrolides can interact with the 50S ribosomal subunit to inhibit protein synthesis, and a mutation of the gene site in the central loop of domain V in 23S rRNA reportedly contributes to the generation of macrolides resistance in *M. gallisepticum*^6^. Additionally, in 23S rRNA, a single site mutation in hairpin 35 of domain II could confer resistance to macrolides^7^. Mutations in the genes encoding ribosomal protein L4 or L22 may also cause resistance by preventing macrolides binding to the ribosome^8^,^9^. In our laboratory, we obtained *M. gallisepticum* strains that were highly resistant to macrolides by using *in vitro* selection, and we identified point mutations in 23S rRNA in macrolide-resistant mutants^10^. Although gene expression has been profiled in the macrolide-resistant and susceptible parent strains, little is known about the global proteome alterations associated with resistance in *M. gallisepticum* mutants. Determining these proteome changes could lead to improved understanding of antibiotic resistance mechanisms.

Here, we used *in vitro* selection and performed comparative proteomics analysis of tylosin-resistant and parent strains of *M. gallisepticum* to investigate the proteome alterations associated with resistance mutations. Our findings indicate that specific enzymatic activity could lead to tylosin resistance in *M. gallisepticum*.

## Results and Discussion

### Selection of Tylosin-resistant Mutants

For the purpose of this study, we defined that the tested strains presented resistance when the minimum inhibitory concentration (MIC)increased ≥8-fold in comparison with the MIC obtained for the corresponding parent strain^11^. After ten passages, the resistant strains could tolerate 512 μg/mL of tylosin, whereas the parent strain (S6) was susceptible to tylosin (MIC = 0.016 μg/mL), tilmicosin (MIC = 0.016 μg/mL), and erythromycin (MIC = 0.125 μg/mL). The resistant strains also exhibited cross-resistance to tilmicosin (MIC = 512 μg/mL) and erythromycin (MIC = 512 μg/mL). The resistance phenotype remained stable in all of the cloned mutants after five consecutive sub-cultures in antibiotic-free medium.

### 2D DIGE Analysis and Identification of Differential Protein Expression

**Figure 1:**
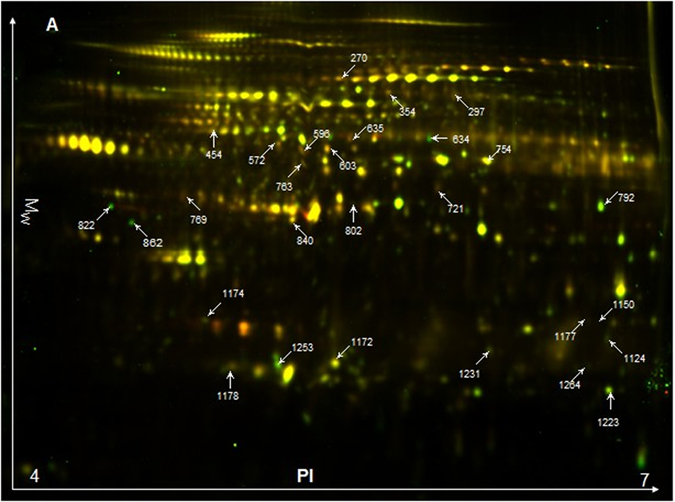
Representative 2D-DIGE proteome map of tylosin-resistant strains vs. parent strains of *M. gallisepticum*. The green spots represent up-regulated proteins, whereas the red spots represent down-regulated proteins (in the resistant strains vs. the susceptible strains).

We analyzed the proteomes of *M. gallisepticum* obtained from tylosin-resistant mutants and their corresponding susceptible parent strains by using 2D-DIGE with the pI range of 4.0–7.0. The representative scanning image of the proteome is shown in Fig. 1. In the images, there were 1400–1600 spots per gel and three replicates showed well-resolved spots and high reproducibility. Following protein identification of potentially interesting spots, we found that 13 proteins were differentially expressed (8 up-regulated and 5 down-regulated) between the mutant and parent strains (Table 1). The matched peptides and spectra are presented in Supplementary Fig. S1). Where individual proteins were represented by multiple spots in the 2-DE gel this may due to posttranslational modification leading to shifts in the gel.

### Validation of Selected Proteins by Western Blot

**Figure 2:**
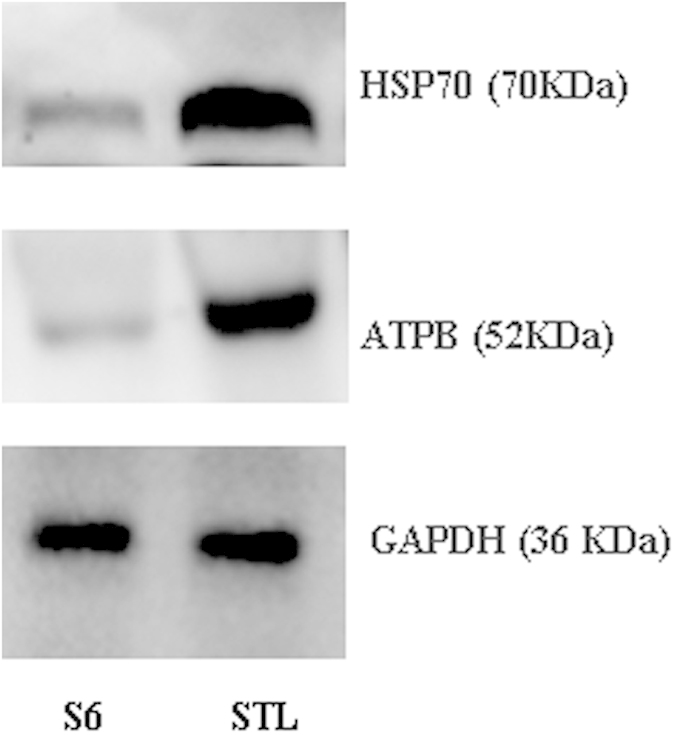
Western blot analysis of DnaK-HSP70 and ATPB in resistant (S-tyl) and susceptible (S6) strains of *M. gallisepticum*.

The protein GAPDH, which did not change significantly between the mutant and parent strains, was used as the internal loading control of the protein concentration in the extracts. The 2D-DIGE protein profiles of DnaK-HSP70 and ATP synthase subunit beta (ATPB) showed that they were more abundant in tylosin-resistant strains. Validation of these proteins by western blot confirmed significant over expression of DnaK-HSP70 and ATPB in resistant strains compared with their corresponding susceptible strains (Fig. 2).

### Validation of Selected Proteins by Multiple Reaction Monitoring (MRM)

**Figure 3:**
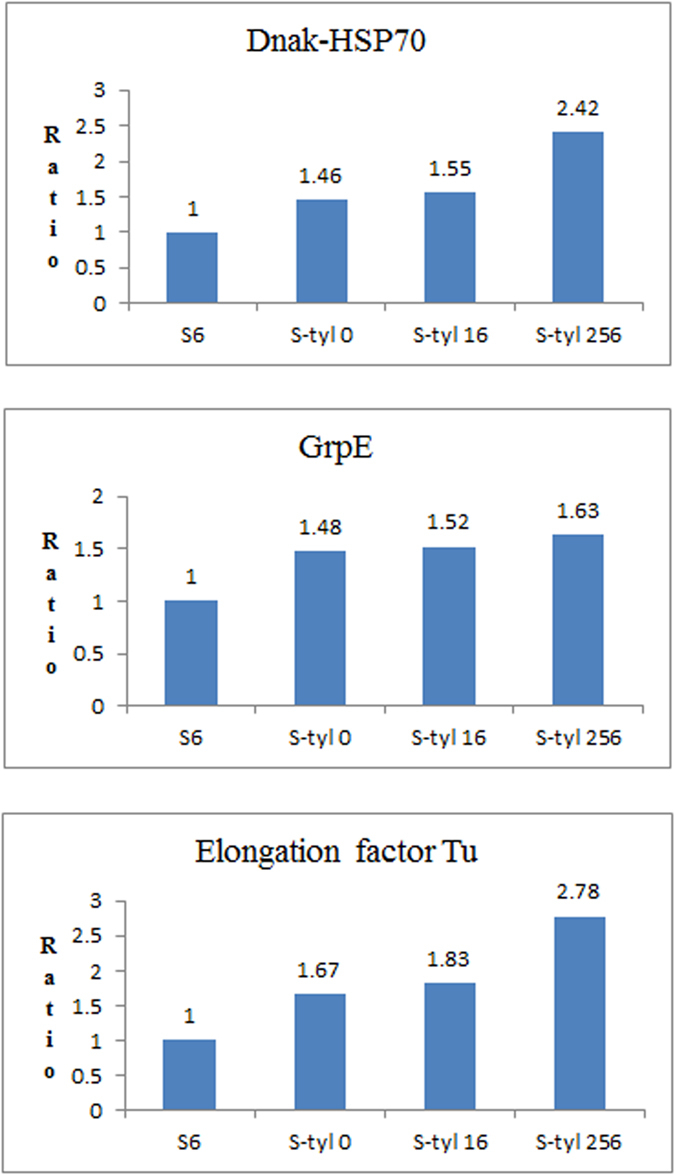
MRM quantitative results.

In order to check the expression of regulated proteins under the pressure of different concentration of tylosin, quantitative analysis of three proteins (DnaK-HSP70, GrpE, elongation factor Tu) was performed by targeted mass spectrometry (MS). The proteins were obtained from susceptible strains cultured without tylosin and resistant strains cultured in a series of concentration of tylosin (0, 16, 256 μg/mL) after five consecutive sub-cultures, and analyzed by nano liquid chromatography-tandem quadrupole MS using MRM technique. As illustrated in Fig. 3, the up-regulation of selected proteins was confirmed.

### Functional Categories and Analysis

We performed protein ontology classification by importing the 13 proteins identified by nano liquid chromatography-quadrupole-time of flight mass spectrometry into the PANTHER HMM sequence scoring system^12^. We identified 12 proteins in the database, with eight proteins annotated in terms of molecular function, biological process, and protein class. In the molecular function and biological process categories, the eight proteins were associated with catalytic activity and metabolic process, respectively. For the protein class category, the proteins were classified into groups associated with nucleic acid binding (25%), oxidoreductase (12.5%), transporter (25%), and transferase (37.5%).

**Figure 4:**
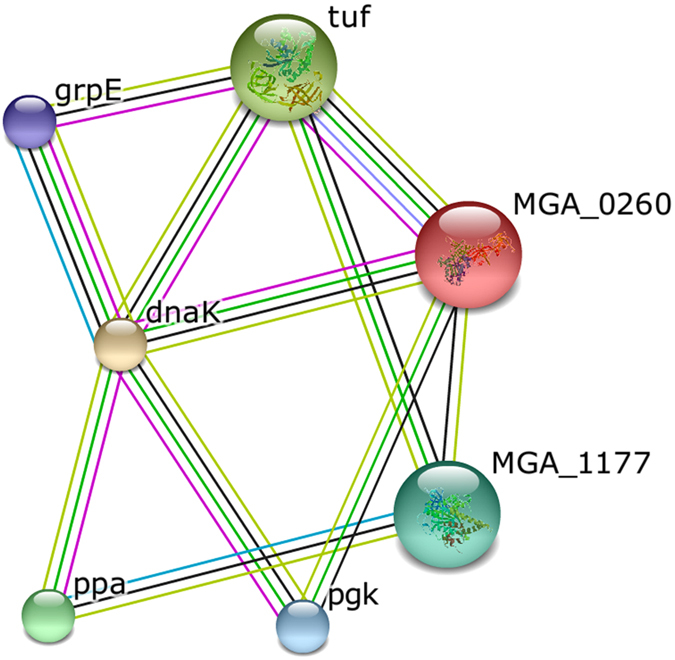
Network of identified proteins, visualized by STRING software (version 9.1). Identifiers: tuf: elongation factor Tu; MGA_0260: elongation factor G; MGA_1177: F0F1 ATP synthase subunit beta; pgk: phosphoglycerate kinase; ppa: inorganic pyrophosphatase.

We investigated the involvement of the identified proteins in protein-protein interaction networks by using STRING 9.1^13^. The results are presented in Fig. 4, where unconnected nodes are removed. Notably, up-regulated elongation factor G and elongation factor Tu were significant nodes in the network. Other important proteins included three up-regulated proteins, ATPB, DnaK-HSP70, GrpE, and two down-regulated proteins, inorganic pyrophosphatase and phosphoglycerate kinase. According to Uniprot annotation, elongation factor Tu promotes the GTP-dependent binding of aminoacyl-tRNA to the A-site of ribosomes during protein biosynthesis, while elongation factor G catalyzes the GTP-dependent ribosomal translocation step. During this step, the ribosome changes from the pre-translocational to the post-translocational state as the newly formed A-site-bound peptidyl-tRNA and P-site-bound deacylated tRNA move to the P and E sites, respectively. Elongation factor G also catalyzes the coordinated movement of the two tRNA molecules, the mRNA, and the conformational changes in the ribosome. ATP synthase subunit beta produces ATP from ADP, and the catalytic sites are hosted primarily by the beta subunits. Inorganic pyrophosphatase catalyzes the conversion of one molecule of pyrophosphate to two phosphate ions. Phosphoglycerate kinase is involved in glycolysis/gluconeogenesis, catalyzing the reversible transfer of a phosphate group from 1,3-bisphosphoglycerate to ADP producing 3-phosphoglycerate and ATP. A decrease in the activity of the glycolytic pathway may place cells under stress to rapidly generate energy in order to carry out necessary cellular functions^14^,^15^. The up-regulation of ATPB and down-regulation of inorganic pyrophosphatase and phosphoglycerate kinase could combine to generate large amounts of energy for protein biosynthesis. Furthermore, another of the up-regulated proteins, bifunctional protein FolD, catalyzes the oxidation of 5,10-methylenetetrahydrofolate to 5,10-methenyltetrahydrofolate and then the hydrolysis of 5,10-methenyltetrahydrofolate to 10-formyltetrahydrofolate; 10-formyltetrahydrofolate is the donor of the formyl groups during the formylation of initiator tRNA in prokaryotic cells.

Crystal structures of the ribosomal subunit complexed with macrolides show that the antibiotics bind in the polypeptide exit tunnel adjacent to the peptidyl transferase center, which suggests that they inhibit protein synthesis by blocking the egress of nascent polypeptides^16^. Given the functions of the proteins identified in our study, we suggest that tylosin-resistant *M. gallisepticum* could have developed resistance in three ways: (i) the production of 10-formyltetrahydrofolate was catalyzed to promote the formylation of the methionyl initiator tRNA; (ii) elongation factors were over expressed, which promoted the binding of tRNA to ribosomes and catalyzed ribosomal translocation and conformational changes; and (iii) different energy-producing pathways were regulated to increase energy production, which would be used for the ribosomal translocation and conformational changes that could overcome the blocking action of tylosin.

## Methods

### Selection of Resistant Mutants and Determination of Minimum Inhibitory Concentration

*M. gallisepticum* ATCC 15302 S6, a reference strain, was used to select tylosin-resistant mutants. The selection of mutant strains and determination of the MIC were performed as described in our previous study^10^. Briefly, the strains were inoculated at 37 °C in mycoplasma broth (MB) medium for 5–7 d until a color change (pink to orange-yellow) was observed. We added 20% (v/v) sterile glycerol to the cultures, and then they were separated into aliquots and stored at −70 °C. To select resistant mutants, we performed serial passaging in MB medium containing a subinhibitory concentration of tylosin. Ten passages were performed, and aliquots of the retained culture were sub-cultured on mycoplasma agar plates to obtain clones for further MIC determination. We determined the MICs of tylosin for the *M. gallisepticum* S6 and *in vitro* induced resistant strains by using the broth dilution method in 96-well microtiter plates. In these plates, each well contained diluted tylosin in the range 0.008–512 μg/mL and an inoculum of 10^5^ color-changing units in 0.2 mL of MB medium. The microtiter plates were incubated at 37 °C and examined daily for 5–7 d. MIC was defined as the lowest concentration of antibiotic that caused a color change in the medium at a time when the growth control (without antibiotic) also showed a color change. To confirm our results, all MIC tests were carried out in triplicate.

### Protein Isolation

For the 2D DIGE analysis, the resistant strain was cultured in MB medium with low concentration of tylosin (16 μg/mL). The cells from 30 mL of culture were washed three times in Tris-buffered saline and suspended in 500 μL lysis buffer (7 M urea, 2 M thiourea, 4% CHAPS, 20 mM Tris-base, 1 mM MgCl_2_, 1 mM PMSF, 5 μL nuclease mix, and 2.5 μL protease inhibitors). Subsequently, the samples were solubilized by sonication on ice for 3 min, and then centrifuged at 40,000 *g* for 30 min at 4 °C. For purification, we applied a 2D clean-up kit according to manufacturer’s instructions (GE Healthcare). The proteins were resuspended in sample solution (7 M urea, 2 M thiourea, 2% CHAPS, 30 mM Tris-base) and adjusted to pH 8.5. The final protein concentrations were determined with the 2D Quant kit (GE Healthcare).

### CyDyes Labeling

Protein samples were labeled with three CyDye DIGE fluors and prepared according to the manufacturer’s protocol (GE Healthcare). Briefly, 50 μg samples of the parent and mutant strain lysates were separately labeled with 400 pmol of Cy3 and Cy5. A pooled internal standard was generated by combining equal amounts of each sample, and then labeled with Cy2. The labeling process was conducted on ice in darkness for 30 min and then quenched by the addition of, and reaction with, 1 μL of 10 mM lysine for 10 min under the same conditions.

## 2D Electrophoresis

We mixed the CyDye-labeled protein samples that were to be separated in the same gel, and 150 μg of these proteins were diluted to a final volume of 450 μL with rehydration solution (7 M urea, 2 M thiourea, 2% CHAPS, 40 mM dithiothreitol (DTT), 0.5% IPG buffer pH 4–7, 0.002% w/v bromophenol blue). Samples were applied to immobilized pH gradient (IPG) strips (24 cm, pH 4–7 NL; GE Healthcare) by in-gel passive rehydration for 16 h at 20 °C. We performed isoelectric focusing using an Ettan III IPGphor system (GE Healthcare) with a voltage gradient increase to 8000 V and a total of 65 kVh at 20 °C. Subsequently, the IPG strips were equilibrated for 15 min in buffer A (6 M urea, 50 mM Tris-HCl pH 8.8, 30% v/v glycerol, 2% w/v SDS, 0.002% w/v bromophenol blue) containing 1% w/v DTT and then for another 15 min in buffer B containing 2.5% w/v iodoacetamide (both steps were conducted with protection from light at room temperature). The second dimensional separation was carried out by using an Ettan Dalt six electrophoresis system (GE Healthcare) on 12% polyacrylamide gels at 15 °C with a program of 1 W/gel for 1 h and then 2 W/gel until the bromophenol blue reached the bottom of the gel. For spot picking and in-gel digestion, we performed a preparative gel loading of 1 mg of unlabeled proteins in parallel and stained with Coomassie Blue G-250^17^.

### Scanning and Image Analysis

The CyDye-labeled proteins in 2D-DIGE gels were visualized using an Ettan DIGE imager (GE Healthcare). Optimal excitation/emission wavelengths for fluorescence detection were 488/520 nm for Cy2, 532/580 nm for Cy3, and 633/670 nm for Cy5. Gel image analysis was processed using DeCyder 7.05 software (GE Healthcare). All protein spots were visually checked and those with volume measurements close to the background and/or with no defined shape, such as precipitated dye, dust particles, and bubbles, were eliminated. Protein spots were detected and in-gel normalized using a differential in-gel analysis module, while a biological variation analysis module was used to automatically match all protein-spot maps in three replicates. Based on the average spot volume ratio, protein spots with at least 1.5-fold changes (p < 0.05) between parent and drug-resistant strains were considered to be significant and transferred to Coomassie-stained preparative gel for spot picking.

### In-gel Digestion

The protein spots of interest were manually excised from the preparative gel, destained with 50% acetonitrile in 50 mM NH4HCO3, and dehydrated in 100% acetonitrile. The gel pieces were reduced in a solution of 10 mM DTT in 25 mM NH4HCO3 for 1 h at 56 °C, alkylated by 55 mM iodoacetamide in 25 mM NH_4_HCO_3_ for 45 min in darkness, and then digested with 10 ng/μL trypsin in 25 mM NH_4_HCO_3_ overnight at 37 °C. The peptides were extracted sequentially with 50% acetonitrile in 0.1% formic acid and 100% acetonitrile. The extracted solution was dried in a Speed-Vac (Thermo Fisher) and resuspended in 10 μL 0.1% formic acid for LC-MS analysis.

### Protein Identification

We identified proteins using a nano Acquity UPLC system coupled to Synapt G2 mass spectrometer (Waters, Milford, MA, USA) with a BEH 130 C_18_ column (75 μm i.d. × 150 mm, 1.7 μm). The mobile phase consisted of solvent A (0.1% formic acid in water) and solvent B (0.1% formic acid in acetonitrile). The flow rate was 0.3 μL/min with a linear gradient at the following conditions: 0–1 min, 99% A; 1–5 min, 99–95% A; 5–35 min, 95–50% A; 35–40 min, 50–10% A; 40–45 min, 10% A; 45–46 min, 10–99% A; 46–65 min, 99% A. The injection volume was 1 μL. The eluting peptides from the nano-LC were ionized (electrospray ionization; capillary voltage 3.0 kV, cone voltage 35 V) and analyzed by MS^E^ mode. The MS data was acquired in the range m/z 100–2000 with a scan time of 0.6 s. Lock mass reference scans were acquired every 30 s using [Glu^1^]-fibrinopeptide B ([M+2H]^2+^, m/z 785.8426). The specific identities of the proteins were confirmed by database searching using PLGS software (version 2.4) (Waters). The peptide spectra were searched against the *M. gallisepticum* S6 proteome database (Uniprot proteome ID: UP000018735). We set the following database search parameters: one miss cleavage was permitted; carbamidomethylation of cysteine as fixed modification and oxidation of methionine as variable modification; each protein identification was determined by at least 7 tryptic fragments and a matching peptide by at least 3 tryptic fragments; mass tolerance was 0.5 Da.

### Western Blot

The proteins were separated on 12% SDS-PAGE gels and electro blotted onto a nitrocellulose membrane. The membrane was blocked with 5% defatted milk for 1 h at room temperature, and then incubated with primary antibodies, mouse anti-ATPB monoclonal antibody 1:100 (Abcam, MA, USA), mouse anti-HSP70 monoclonal antibody 1:1000 (Beyotime Biotech, Shanghai, China), and mouse anti-GAPDH monoclonal antibody 1:2000 (ZS Biotech, Beijing, China), overnight at 4 °C. Excess antibodies were removed by washing with Tris-buffered saline and 0.05% Tween 20, followed by incubation with secondary antibody (goat anti-mouse IgG conjugated with horseradish peroxidase, 1:3000) at room temperature for 2 h. The signals were detected by using the electrogenerated chemiluminescence method.

### Multiple Reaction Monitoring

Four groups of strains, including S6, resistant strain cultured without tylosin (S-tyl 0), resistant strain cultured with 16 μg/mL of tylosin (S-tyl 16), resistant strain cultured with 256 μg/mL of tylosin (S-tyl 256), were involved in this validation study. The proteins were digested based on FASP protocol^18^. Briefly, 4 pmol of BSA as internal standard was equally spiked in each sample with 200 μg of MG proteins. The proteins were reduced in 8 M urea, 20 mM DTT buffer at 37 °C for 60 min, and alkylated in 60 mM IAA buffer in the dark for 30 min. The mixtures were then transferred to ultrafiltration unit (10KD MWCO) and centrifuged at 14,000 g for 20 min, followed by buffer exchange processing with 200 μL of 25 mM NH_4_HCO_3_. 4 μg of trypsin in 100 μL of 25 mM NH_4_HCO_3_ was added to the ultrafiltration unit and incubated at 37 °C over night. Peptides were eluted into the collection tube by centrifuging at 14,000 g for 20 min. Additional 100 μL of 25 mM NH_4_HCO_3_ was added to wash the ultrafiltration unit membrane and centrifuged at 14,000 g for 20 min.

The MRM experiment was conducted with an Eksigent Nano LC and a QTRAP 6500 mass spectrometer system. The mobile phase consisted of solvent A (2% acetonitrile and 0.1% formic acid in water) and solvent B (2% water and 0.1% formic acid in acetonitrile). The trap column (200 μm × 0.5 mm) and analytical column (75 μm × 150 mm) were packed with 3-μm ChromXP C18-CL resin (Eksigent cHiPLC columns). The injection volume was 5 μL. The peptides were separated with a gradient from 5% to 20% B over 50 min, to 32% B over 20 min, and to 80% B over 5 min, at a flow rate of 0.3 μL/min. The column was then flushed with 80% B for 5 min, and re-equilibrated with 5% B for 10 min. For MS settings, the scheduled MRM type was selected with the imported MRM transition list. Other MS parameters were as follows: MRM detection window, 600 s; target scan time, 2.5 s; ion source gas 1, 15; curtain gas, 30; ionspray voltage floating, 2400. All the data were acquired by Analyst v1.6 and processed with MultiQuant™ v3.0.

## Additional Information

**How to cite this article**: Xia, X. *et al.* Proteomic analysis of tylosin-resistant *Mycoplasma gallisepticum* reveals enzymatic activities associated with resistance. *Sci. Rep.* **5**, 17077; doi: 10.1038/srep17077 (2015).

## Supplementary Material

Supplementary Information

## Figures and Tables

**Table 1:** Differentially Expressed Proteins Identified by nano LC-MS After 2D-DIGE Analysis.

Entry	MasterNo.	Protein Description	Protein ID	MW (Da)	PI	ProteinScore^a^	Coverage(%)	AverageRatio	T-test
Up-regulated proteins in tylosin-resistant strains									
1	270	Elongation factor G	V5W3E9	76696.5368	5.4	1288	33.2	3.74	0.037
2	769	DnaK-HSP70	V5W237	64514.8049	5.1	2080	24.2	1.84	0.046
3	1150	Bifunctional protein FolD	V5W2P5	32223.4106	7.9	2961	30.0	2.26	0.016
4	1253	Trigger-factor-like protein	V5W187	24231.0354	5.1	7668	74.7	1.5	0.043
5	603	F0F1 ATP synthase subunit beta	V5W3S8	52077.9423	5.2	237	15.4	1.93	0.049
6	596	ATP synthase subunit beta	V5W1F1	51492.1073	4.9	34298	84.0	2.46	0.047
7	840	Elongation factor Tu	V5W1B4	43099.2737	5.5	1002	27.4	1.83	0.0095
8	721	GrpE	V5W1Q0	39519.2692	5.8	2449	33.7	2.59	0.0014
Down-regulated proteins in tylosin-resistant strains									
9	1124	Transcription termination factor NusG	V5W2E3	30114.2862	6.1	15136	79.7	2.83	0.044
10	1264	2-C-methyl-D-erythritol 4-phosphate cytidylyltransferase	V5W1D5	27278.5366	6.4	235	17.3	1.7	0.04
11	634	Phosphoenolpyruvate-protein phosphotransferase	V5W177	64566.7238	5.6	254	10.7	3.37	0.017
12	1223	Inorganic pyrophosphatase	V5W1C8	21128.6363	5.9	1877.	23.7	2.16	0.046
13	792	Phosphoglycerate kinase	V5W1F6	45537.1825	6.7	16675	58.8	2.34	0.0017

^a^Score given by PLGS software.
