# Supplementary material for: Proteomic analysis of tylosin-resistant Mycoplasma gallisepticum reveals enzymatic activities associated with resistance
Source: Sci Rep. 2015 Nov 20;5:17077. doi: 10.1038/srep17077 (PMC4653647; doi:10.1038/srep17077)
Supplement: Supplementary Information [file srep17077-s1.pdf]

Supplementary information

Proteomic analysis of tylosin-resistant *Mycoplasma gallisepticum* reveals enzymatic activities associated with resistance

Xi Xia[1,3], Congming Wu[2,3], Yaowen Cui[3], Mengjiao Kang[4], Xiaowei Li[2,3], Shuangyang Ding[1,3], Jianzhong Shen[1,3]*

[1] Beijing Key Laboratory of Detection Technology for Animal-Derived Food Safety, China Agricultural University, Beijing 100193, People's Republic of China

[2] Key Laboratory of Detection for Veterinary Drug Residue and Illegal Additive, Ministry of Agriculture, Beijing 100193, People's Republic of China

[3] College of Veterinary Medicine, China Agricultural University, Beijing 100193, People's Republic of China

[4] China Institute of Veterinary Drugs Control, Beijing 100081, People's Republic of China

*Corresponding author. Tel: +86-10-6273-2803; Fax: +86-10-6273-1032; E-mail: sjz@cau.edu.cn

Figure S1. Matched peptides and spectra of the identified proteins

Elongation factor G

```
         10         20         30         40         50
MARQYPLEKF RNFGIMAHID AGKTTTSERI LFHSGKTHKI GETHDGASVM
         60         70         80         90        100
DWMAQEKERG ITITSAATSV TWKDCQLNLI DTPGHVDFTV EVERSLRVLD
        110        120        130        140        150
GAVAVLDAQM GVEPQTETVW RQASRYEVPR IVFVNKMDKT GANFQRSVDS
        160        170        180        190        200
IHSRLGVKSV PIQLPIGAEN DFVGIIDLVE MKAYFFDGGE NENYETKEIP
        210        220        230        240        250
AEYLEEAKKA HNHMLDEIVT FDEAVMEKYL DGQEISKAEI KSCIRKGVVS
        260        270        280        290        300
STLFPVLCGT AFKNKGVKPL LDAVVDYLPS PIDVPPAKGY KVGEEVQIPT
        310        320        330        340        350
SDDAPFVGLA FKVATDPFVG RLTFVRVYSG ILTSGSYVIN TTKDKKERVS
        360        370        380        390        400
RIVKMHAQQR DEIDEIRAGD ICAIVGLKDT TTGDTIASEN QNLTLESMTF
        410        420        430        440        450
AQPVISLAVE PKTKADQEKM GISLSKLAEE DPTFRTYTDE ETGQTIIAGM
        460        470        480        490        500
GELHLDILVD RLRREFKVDV NVGAPQVSYR ETLKAKADVE GKYIKQSGGR
        510        520        530        540        550
GQYGHVVITF EPNHDKGFEF EDKIVGGKIP KEYIKSVKAG LEAAMNNGPL
        560        570        580        590        600
AGYPMIDIKA SLFDGSYHDV DSNEMAYKIA ASMALKEAGK RCQPALLEPI
        610        620        630        640        650
MAIEVTVPEQ YFGDTMGDIS SRRGMIEGTE SRDNVQVIKA KVPLSEMFGY
        660        670        680        690
ATDLRSFTQG RGNYIMQFSH YAEAPKSVVE KVIEDKAKKN KTA
```

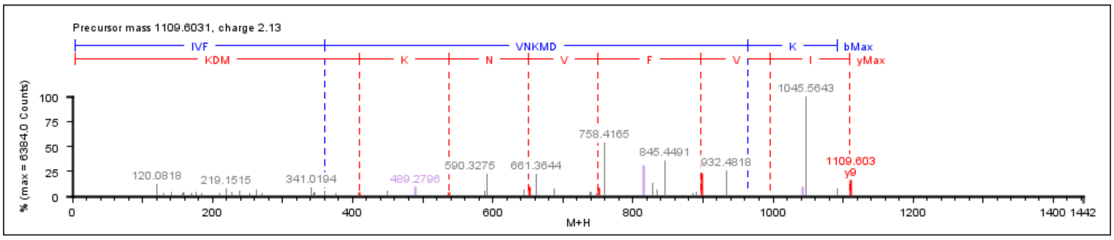

DnaK-HSP70

```
         10         20         30         40         50
MSNNNGLIIG IDLGTTNSCV SVMEGTQKVV IENPEGKRTT PSVVSYKNGE
         60         70         80         90        100
IIVGDAAKRQ MLTNPNTIVS IKRLMGTSKK VKINDKGVEK ELTPEEVSAS
        110        120        130        140        150
ILSYLKDYAE KKTGQKISRA VITVPAYFND AERQATKTAG KIAGLTVERI
        160        170        180        190        200
INEPTAAALA YGIDKGHREM KVLVYDLGGG TFDVSLLDIA DGTFEVMATA
        210        220        230        240        250
GDNRLGGDDW DNKIIEWIIA EIKKDHPSLD LKSDKMAMQR LKEAAERAKI
        260        270        280        290        300
ELSAQLETLI SLPFIAVTPE GPVNAELTLS RAKFEELTKD LLERTRNPIA
        310        320        330        340        350
DVLKEAKVDP SQVDEILLVG GSTRMPAVQK LVESMIPNKT PNRTINPDEV
        360        370        380        390        400
VAIGAAVQGG VLRGDVKDIL LLDVTPLTLA IETLGGVATP IIKRNTTIPV
        410        420        430        440        450
SKSQIFSTAQ DNQESVDVSI YQGERPMARE NKSLGTFSLG GIQPAPKGKP
        460        470        480        490        500
QIEITFNIDA NGILNVKAKD LTTGKENSIT ISNSSELDEN EIQRMIRDAE
        510        520        530        540        550
ANKERDAIVK QRIEMRYEGE GIVNTINEIL GSKEAEALPA QEKASLTKIV
        560        570        580        590
DGINGALKAE KWDELKEQID GFKKWRDDMS KKYGGGEAPA EPK
```

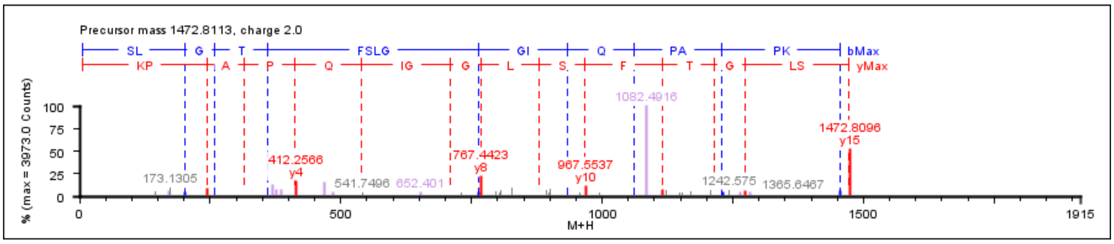

Bifunctional protein FolD

```
         10         20         30         40         50
MFIKLDGTKL SQKLKEDLAK KVNNQKIKLL IIISDPSEAS RIYVRNKINY
         60         70         80         90        100
CESLGIQTEV YDLSKIDNTN QFIVKMNQKI SLSNPNGVLV QLPIKERLDT
        110        120        130        140        150
NKIIENIPID LDVDAFLYHR FDQDQKEKVI PCVLNAVLEL FKEYQLSFLD
        160        170        180        190        200
KKILLIGNGI TANQPIVNYL NEHQINFDLI TKENSQLLEE KTKVADLVIS
        210        220        230        240        250
AVGKAKFLGN YEFKQGVIFI DIGIDKYFDP EQSKYLVCGD FDYDKLKEIA
        260        270        280
SYGTPTPGGI GPLTIYSLVK NLINLSEIQK VNK
```

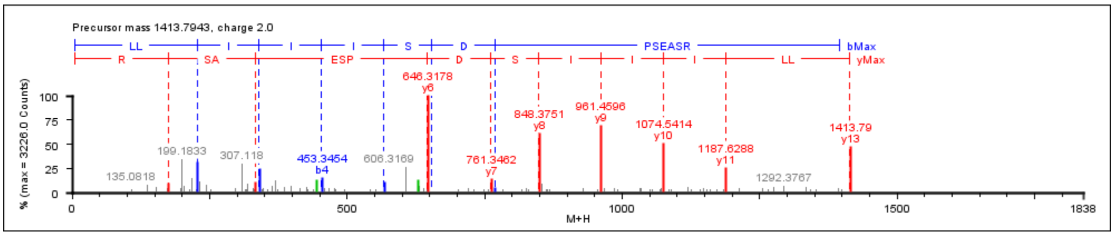

Trigger-factor-like protein

```
         10         20         30         40         50
MSSKLKLIKP IDYSKEIKIT QFFIDPAMME QQRQRIKAAL PKEMNDETMM
         60         70         80         90        100
QYELLQLSIK DNVFSAIMNY LAEHFEFEID QAEVKKLIEQ LKASGLGAQR
        110        120        130        140        150
EELLANMADK IIKKGLMFDY LSEQWKVKVS DNEVKNMLDI YYEKTNQSIH
        160        170        180        190        200
DVLNDQQKFE SVRSSIFEEK MVLKTISMFL IRFNMQNPNY VEESETDKSS

EPKSIN
```

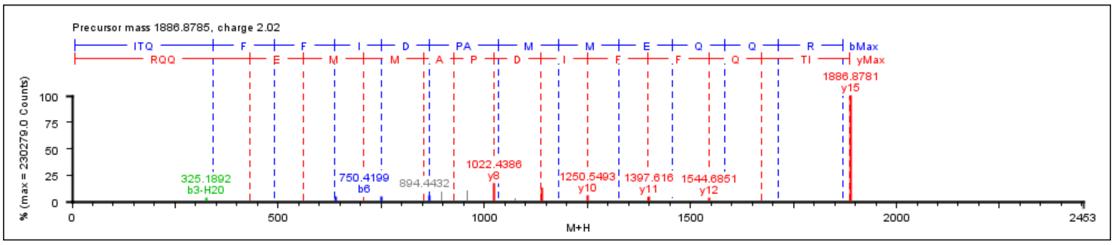

F0F1 ATP synthase subunit beta

```
        10         20         30         40         50
MIGHITKIWN NVVEATFKKE ELPKVDFILT LHNNTCVLLV KRIIDDTSVR
        60         70         80         90        100
AIVIYQKQSI KIGDQVVNTN DTLKVPVGQA AKNNIYDISG VPMLQQRAKG
       110        120        130        140        150
IKYIEMNSTI RKEKKIYSKH EILETGIKAI DFFMPILKGH NLGILGGAGV
       160        170        180        190        200
GKTVLMKEII FNSSRSNSAK KNSSIFIGSG ERSREGVELY QELEQSNLMK
       210        220        230        240        250
DSMMFISKMD EPPGARMSIV PYGITAAEYL RDVEKEDVLL FIDNIFRFIQ
       260        270        280        290        300
AGNEVAPGLN KKPITGGYQA TLDTEVSNVE DRLYATEDGS ITSFQTLFLP
       310        320        330        340        350
MDDLADPSAV SIFSNLDGSL VLSREQTSKN IFPAFDPLAS SSSSVSPDII
       360        370        380        390        400
GQRHYNAIIE VKSILQKYRD LEDVILILGI DELDKDNKII VKKALQLQNF
       410        420        430        440        450
FTQNFFVAEA YTKAKGVYVP LEETLESVIR IVEGKYINQS PEIFSFIGSN
       460
LNLPTDEELN LNKVDL
```

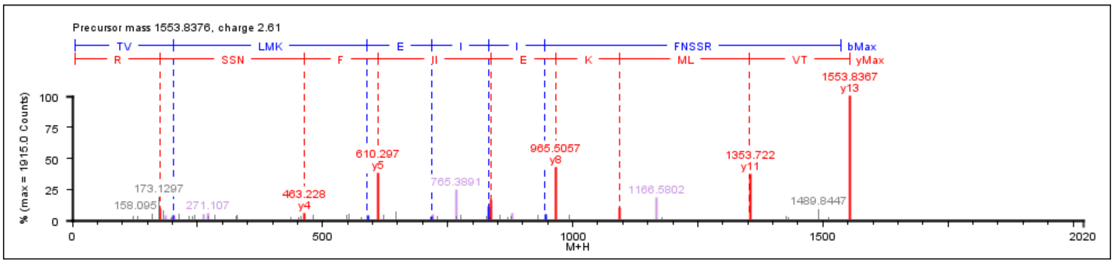

ATP synthase subunit beta

```
        10         20         30         40         50
MNTKYSYGKV YQVVGPVVDV VFEKQDDLPK IYDCLIIDEP NMKLHLEVAQ
        60         70         80         90        100
LIGDDIARCI AMGPTEGLAR NVKVTSTNQP ISVPVGTEVL GRMFNVIGEP
       110        120        130        140        150
IDEKKPIDAS VKRMSIHRPA PSFADQSNEL EIFETGIKVI DLLIPYAKGG
       160        170        180        190        200
KIGLFGGAGV GKTVLVQELI HNIATGHGGL SVFAGVGERT REGNDLYYEM
       210        220        230        240        250
IEGGVIDKTA LVFGQMNEPP GARMRVALTG LTMAEYFRDV NNQDVLLFID
       260        270        280        290        300
NIFRFTQAGS EVSALLGRMP SAVGYQPTLA EEMGSLQERI TSTKSGSITS
       310        320        330        340        350
VQAIYVPADD LTDPAPSTTF THLDAKTVLD RNIASLGIFP AVNPLDSTSR
       360        370        380        390        400
LLDPSVVGIQ HYQTARKVQM ILQKFLELQD IIAILGIDEL SEEDKLTVSR
       410        420        430        440        450
ARKIRNFLSQ PFFVAEKFSG NKGKYVPISE TIKGFSEIVE GKHDDLPEQA
       460        470
FFYVGSIDEA IERAKTLTCN G
```

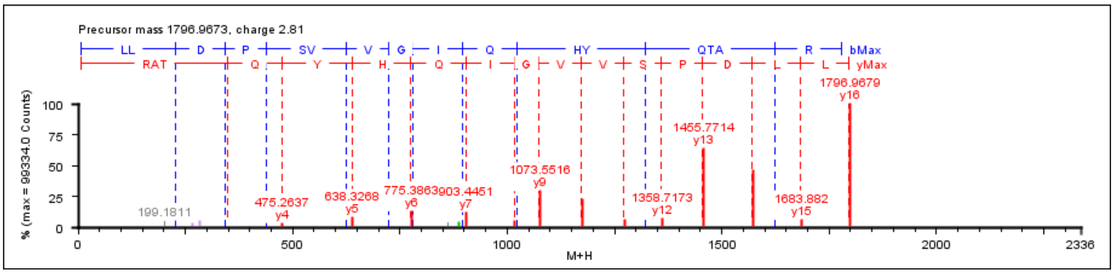

Elongation factor Tu

```
        10         20         30         40         50
MAKERFDRSK PHVNIGTIGH IDHGKTTLTA AICTVLSKAG TSEAKKYDEI
        60         70         80         90        100
DAAPEEKARG ITINTAHVEY ATQNRHYAHV DCPGHADYVK NMITGAAQMD
       110        120        130        140        150
GGILVVSATD GPMPQTREHI LLARQVGVPK MVVFLNKCDV ADDPEMQELV
       160        170        180        190        200
EMEVRDLLKS YGFDGDNTPV IRGSALGALN GEPAWEEKIH ELMKAVDEYI
       210        220        230        240        250
PTPDREVDKP FLLPIEDTMT ITGRGTVVTG RVERGQLKVG EEVEIVGITD
       260        270        280        290        300
TRKVVVTGIE MFRKELDAAM AGDNAGILLR GVDRKDVQRG QVLAKPGSIT
       310        320        330        340        350
PHKKFRAEIY ALKKDEGGRH TAFLNGYRPQ FYFRTTDVTG SIQLKEGTEM
       360        370        380        390
VMPGDNTEII VELISSIACE KGSKFSIREG GRTVGAGTVV EVLE
```

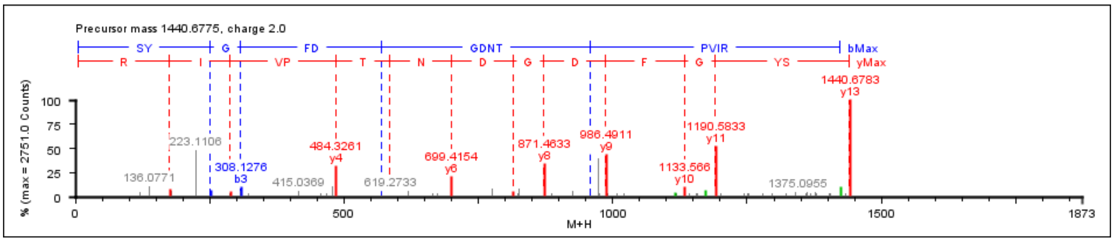

GrpE

```
        10         20         30         40         50
MLNMNNQEHK NQNEEVQKEV KENPAQEEVS QPSDSSNPTS ESAPTLNAQD
        60         70         80         90        100
PNNINLANEN IQIKSSLDDL KNFVQNPKSQ NQKIKAVNQL MYNEFVKIET
       110        120        130        140        150
AINNINNYIE NLTHRLELTN ENFKTKVQEV ESKAQQKIND RIEELDKRKK
       160        170        180        190        200
EEIENAKKYA IEKSIDSAIN IVDQLEIALE FASLDPAVKN YVSGFKMVLN
       210        220        230        240        250
SFVNWLASVN IHRMDIKPGD KFDEKYMSAS DKASDPDYPA DHVCKVMKSG
       260        270        280        290        300
YKLYDRVVRH AMVAVSDGVG YVEPASSEQE QPQTPAKSTQ TPPAQETASA
       310        320        330        340        350
PAKTKPTAPP TNNLNANKPP VVSNPPHQKP VQPPQSNTPG PKQPITHQVI

KKS
```

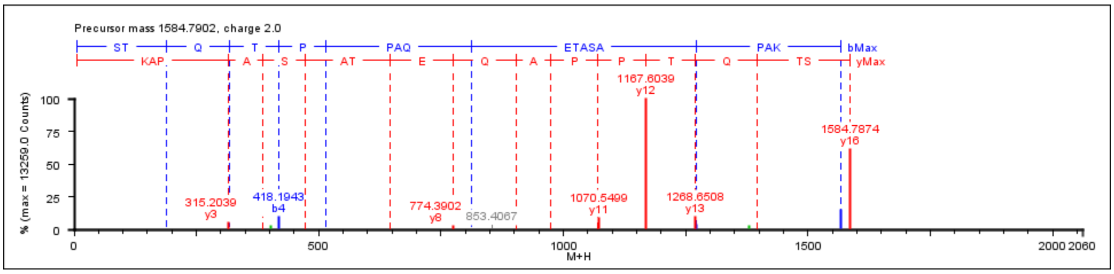

Transcription termination factor NusG

```
         10         20         30         40         50
MSKKSTAQWY IATTTNGNED SVIKTLKAKV RALHFEDQIL DCKVIKFRSV
         60         70         80         90        100
EETIFDSNNP THNIPSTMRN STYIKWVTID NGVYKKYKIT DTNKYPGYIY
        110        120        130        140        150
IKMEMNEAAW FAVRNTVNIT GIVGSSGKGA KPIPISSSEE LDLLNGESFD
        160        170        180        190        200
QNYRIVITPN AIIEMDRNLF NERGELILDE NTAKTIVHKK KSDSADKYGD
        210        220        230        240        250
MSTEKEQVDE AIELKVGHMV DINSGDYSGL SGQISRIIDN DEYIVDVQIL
        260
GKLVSVKLNK KQLKLSV
```

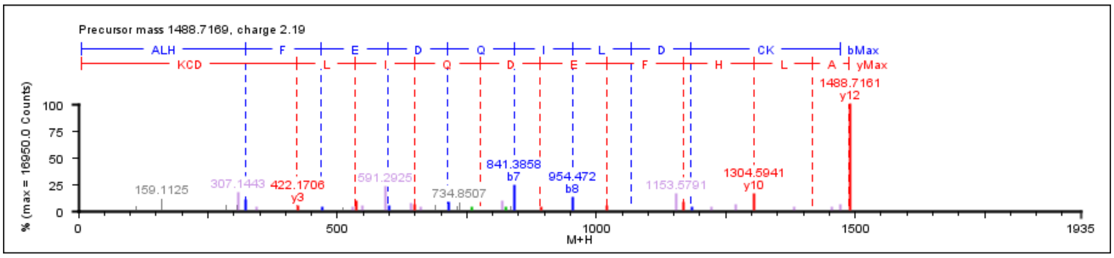

2-C-methyl-D-erythritol 4-phosphate cytidylyltransferase

```
        10         20         30         40         50
MITVKMITKT VGIILASGSS SRLGLTDQLK QFYLVNNKMV YEYSLDQFLA
        60         70         80         90        100
SNLFDLIYLV VNEQHLEMIK TKHSDNHLIK VIAGSKINRH LSFIHAINDM
       110        120        130        140        150
IACDYNDQTK IVVHDSARPN INQSILSDCL NALDDYQCVS LYQDIASSLI
       160        170        180        190        200
KIDQINQSQD HLDRDQIKMI YTPQATWLKT VKDLVDDQQN HLDLTDFLLK
       210        220        230
LKGVKAHWIK TKIDCFKITT LADLEHFKLL VNNRKDE
```

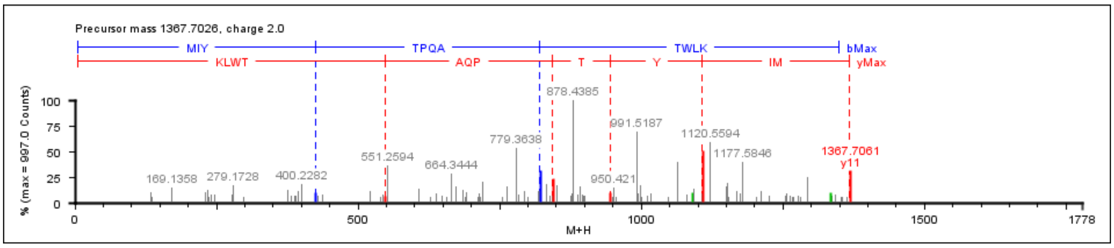

Phosphoenolpyruvate-protein phosphotransferase

```
        10         20         30         40         50
MKKYEGIGAS NGVAIAKAYV LKAPVFNFDN NKIKPTEVED TITKIKQAFD
        60         70         80         90        100
KSSEQLRELR TIALKNLGEE IAVVFDGHIN IVNDPMLFDQ ISQEVKEHLA
       110        120        130        140        150
NAPTAVSKIY DQTKAMFEGI EDVYLKERAS DIGDVKKRIL SNLLNVALPD
       160        170        180        190        200
LLAINQEVII IADDLTPSET SLLNKQYVKG FATNIGGRTS HSAIMARTLE
       210        220        230        240        250
IPAVLSLKTI TETLKHDQLI CIDGNKGVVY SDLSEADITS LKQDQQRYNE
       260        270        280        290        300
SQAKLKKYLP PKAVTLDGHE TIVAVNIGKP IDLLKGNDYG AKGVGLFRTE
       310        320        330        340        350
FLYMDSANWP DEETQFNAYK QALDYANNET VIIRTLDIGG DKKLNYYQFP
       360        370        380        390        400
EEMNPFLGFR AIRFTNQNPD IFKAQLRALL RAAKFGSLGI MFPMIANLEE
       410        420        430        440        450
LFKAKEILEE AKKELDQRKV EYGKPLVGIM IEIPSAAVMS DVLAKYVDFF
       460        470        480        490        500
SIGTNDMIQY SFAVDRMSKD VNYLYQPLNP ALLRIVKMTI DGGLKHNVWT
       510        520        530        540        550
GMCGEMAGEP LAIPILLGMG LKEFSMSASS MLKAKELINN LKYSDCKELV
       560        570
NQVINLESEE EVVKKVKEFL AKNNLSVA
```

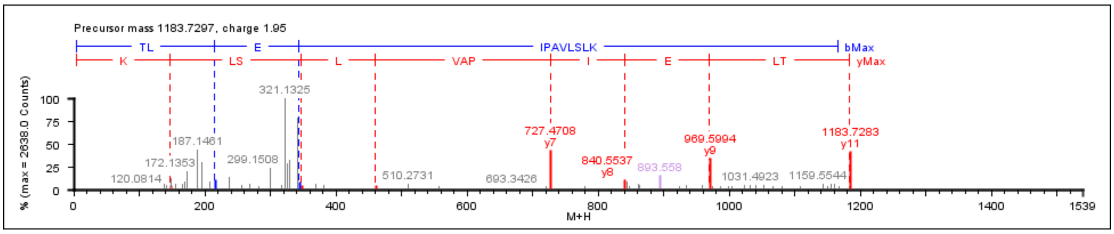

Inorganic pyrophosphatase

```
         10         20         30         40         50
MSNLLELKVT IEIPKGSNIK YEYDRKTGQI SVDRILYGSE VYPHNYGFIK
         60         70         80         90        100
EALDWDGDEL DCLVVANQAF QPGVVVPVRI LGMMGMVDSG ETDNKLIGVI
        110        120        130        140        150
ACDKRFENIR SLKDLGAHAL KEIKGFFETY KLLQNKKVLV KGFKELDSAI
        160        170        180
HEYKNCVSLM NQYGSLPKDE FIAKMKKMYP EKYME
```

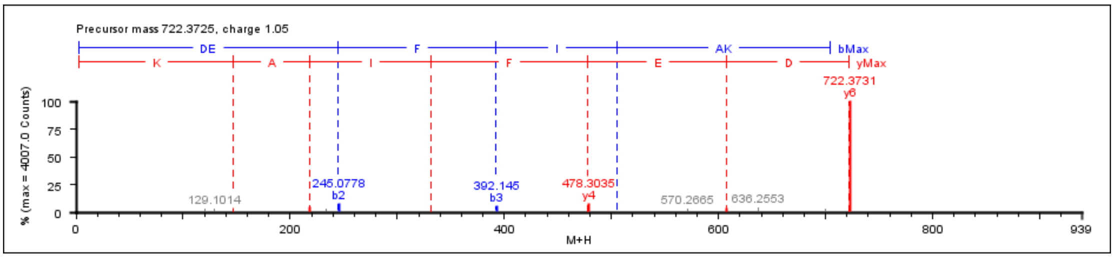

Phosphoglycerate kinase

```
         10         20         30         40         50
MINYNKKTLK DVDLKDKTVI VRVDFNVPIK DNKVIDDTRI VQALDTIKYL
         60         70         80         90        100
IEQNCKIVLL SHLSRIKSLE DISSKKKSLR PVYENLKTKL NNVKFLEENV
        110        120        130        140        150
GYDVVEAVKQ LKHQEVLLLE NTRYNDVDNQ GEVVKKESKN SPELGRFWAS
        160        170        180        190        200
LADVFVNDAF GTSHRAHASN VGIAANISQS CIGFLVQKEL EALSKLTNNP
        210        220        230        240        250
QRPFVVILGG AKVSDKLKVI ESLLKSADHI LIGGGMVNTF NMAKGYHIGK
        260        270        280        290        300
SLFEPEMLET AKKILAEDKD NKIILATDQM VTKASTITDI KTAPAGKCVF
        310        320        330        340        350
AKDEAENEDF EALDIGDESI KTFKSYIAKA KSIFWNGPLG VFENPNYERG
        360        370        380        390        400
SYEIAKAISE SDSYSVIGGG DSAAAANQFK LADKFSFVST GGGASLTFME
        410
QTVLPGIEAI QSK
```

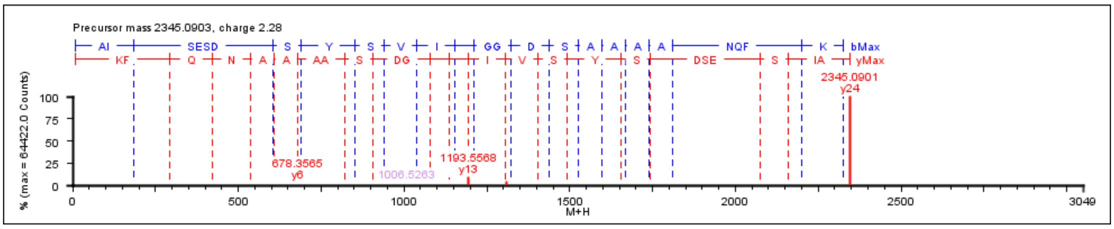

Figure S2. Western blot results

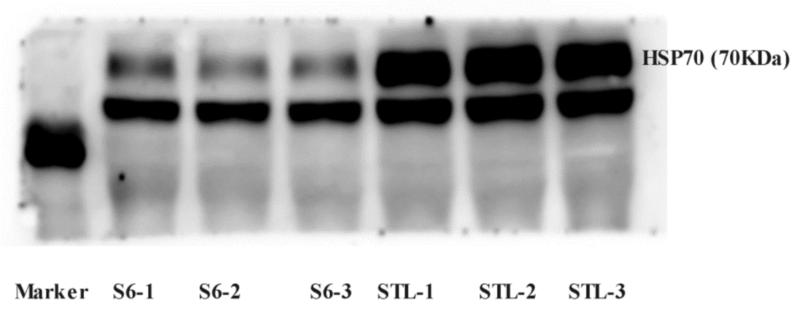

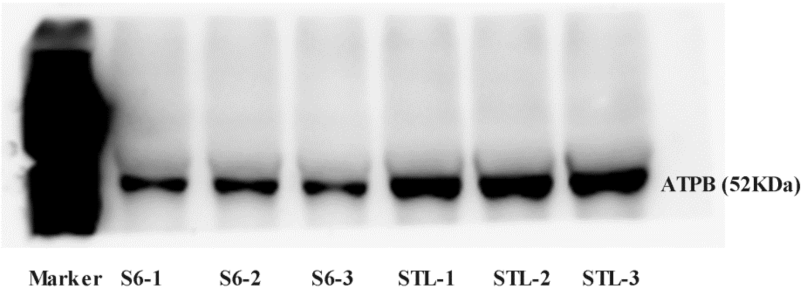

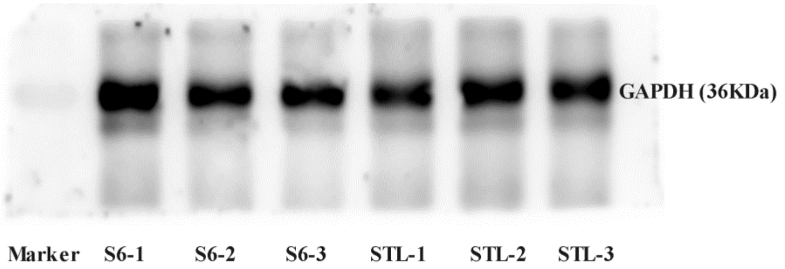
GAPDH (36KDa)
Marker
S6-1
S6-2
S6-3
STL-1
STL-2
STL-3
